# The Management of Necrotizing Gingivitis in Paediatric Patients: A Scoping Review and Two Case Reports

**DOI:** 10.3390/children11081019

**Published:** 2024-08-21

**Authors:** Massimiliano Ciribè, Erika Cirillo, Paolo Giacomo Arduino, Alessandra Putrino, Martina Caputo, Simona Zaami, Gaia Bompiani, Angela Galeotti

**Affiliations:** 1 Dentistry Unit, Management Innovations, Diagnostics and Clinical Pathways, Bambino Gesù Children’s Hospital, IRCCS, 00165 Rome, Italy; 2 CIR—Dental School, Department of Surgical Sciences, University of Turin, 10126 Turin, Italy; 3 Department of Anatomical, Histological, Forensic and Orthopedic Sciences, Sapienza University of Rome, 00161 Rome, Italy; simona.zaami@uniroma1.it; 4 U.N.—E.U. International Research Project on Human Health, Oral Health Section, 1200 Genève, Switzerland

**Keywords:** necrotizing gingivitis, paediatric, antibiotic, ozone, ozone therapy

## Abstract

Necrotizing gingivitis (NG) is an acute inflammatory process with an estimated prevalence of less than 1%. The treatment of choice is usually antibiotics in addition to periodontal treatment. This scoping review aims to detail extent and type of proof related to NG in paediatric patient; moreover, a decision tree protocol was developed to define NG management in paediatric patients based on the presence or absence of systemic compromission. In addition, we also propose the use of ozone treatment as an adjuvant therapy. Seven papers (3 case reports, 2 guidelines, and 2 reviews) were selected for evaluation by reading the full texts. This review outlines the lack of research on the treatment of NG in paediatric patients; we, however, demonstrate the efficacy of the decision tree protocol by describing two case reports in which patients were treated with antibiotics according to the presence or absence of systemic involvement through the implementation of an individualized therapeutic approach, with periodontal ozone therapy. Moreover, the supportive use of this molecule in the management of NG can be a valuable tool in the healing of gingival tissues.

## 1. Introduction

According to the new Classification of Periodontal and Peri-Implant Diseases and Conditions [1], necrotizing gingivitis (NG) is included in the necrotizing periodontal diseases (NPDs), a set of manifestations characterized by the necrosis of gingival or periodontal tissues, pain, and bleeding. These states are sometimes linked to alterations in the host’s immunological condition [1].

NG is an acute inflammatory process characterized by the ulceration and/or necrosis of the interdental gingiva; bleeding; and intense local pain. Removable pseudo-membranes, regional lymphadenopathy, fever, halitosis, and sialorrhea may also occur. It is the most frequent form of NPD, with an approximate worldwide rate of no more than 1%. Young adults (18–30 years) are the population most affected by this condition [2], with an equal distribution between both sexes in Western countries [3].

NG is an infection based on the presence of opportunistic bacteria with spirochetes and Gram-negative bacteria. They include many species: *Bacteroides intermedius*, *Fusobacterium* spp., *Treponema* spp., *Selenomonas* spp., and *Prevotella intermedia* [4,5]. In less developed countries, NG is usually associated with immunosuppression, most notably in relation to HIV, and other conditions such as poor oral hygiene, severe malnutrition, poor eating habits, psychological stress, bad/poor sleeping habits, and tabagism [6]. In developed countries, NG is linked to populations with a poor diet, such as that often observed in college students [7], as well as those with tobacco smoking habits and poor oral hygiene [8]. To date, NG does not seem to be associated with orthodontic treatments, but orthodontic patients could be at higher risk due to the augmented plaque retention on orthodontic devices [9].

The diagnosis of NG mainly occurs when the necrosis is limited to the interdental papilla, showing the typical “punched-out” look [6]. At this stage, the condition can be reversed by treatment, because there is no loss of periodontal attachment. If NG is untreated, it may lead to rapid bone loss; this stage is better known as necrotizing periodontitis. Though uncommon, if necrosis extends beyond periodontal tissues, osteonecrosis and bone denudation may occur (necrotizing stomatitis), although this is currently endemic only in some African countries, while it disappeared in the 20th century in Western countries; these diseases are all stages of the same condition [2]. Therefore, it is important to treat NG promptly; this prevents the disease from progressing [10].

The treatment of NG should be carried out in steps, due to the intense pain of patients and of risk of recurrence. Initially, the acute phase should be managed, then any previous conditions should be treated, such as the disease’s consequences, and at the end, a supportive or maintenance phase should be started [5]. Generally, the managing of NG is mainly local and includes mechanical debridement (if necessary, under local anaesthesia) [4,10,11,12,13] and the use of an antiseptic solution, like chlorhexidine mouthwash [3,9,10,12,13,14,15,16,17,18,19,20] or hydrogen peroxide, sometimes diluted in water [4,10,13,21,22,23]. Providing oral hygiene instructions to patients is necessary [11,13,23,24]. The presence of systemic signs and symptoms (fever, malaise, and lymphadenopathy) should encourage the use of systemic antimicrobials. Its activity against anaerobes makes metronidazole the gold standard for the treatment of NG [6,9,18,23,24,25,26]. Its combined use with amoxicillin can be helpful in aggressive oral infections [25,26]. Systemic drugs like clindamycin, penicillin, and tetracyclines have shown acceptable results too [6,25]. The treatment of NG is similar in adult and young patients because in the literature there is a lack of high-quality research available regarding NG, especially for paediatric patients, for whom the literature typically adapts the adult treatment protocol [27].

Apart from conventional treatments, a therapy based on the application of ozone has found use as an adjuvant therapy for periodontal and mucosal diseases. Ozone (O_3_) is a triatomic compound of three oxygen atoms [28]. Ozone acts like a potent oxidant even though it is not a radical molecule [29]. Its reactions with phospholipids and lipoproteins lead, respectively, to the disruption of bacterial cell walls and cell membranes. Its capacity to stimulate blood circulation increases the red blood cell glycolysis rate. It induces an increase in the amount of oxygen released to tissues. Moreover, if administered in specific concentrations (around 30–55 μg/cc), ozone induces an activation of the immune response and an overall increase in interferon production [30,31,32,33]. These biological mechanisms make ozone therapy applicable in dentistry [30].

One of the peculiarities related to ozone molecules is that, being an unstable gas, it cannot be stored. The medical application of ozone requires the presence of an ozone generator: a high-voltage gradient of 5 to 13 mV, through pure oxygen passes, generates a gas mixture containing 5% ozone and 95% oxygen [31,33]. To date, ozone therapy has been widely investigated for the treatment of different oral conditions [34]; for example, some researchers have studied the clinical effects of ozone during non-surgical treatment of periodontitis, although the results are not always consistent [35]. Many clinicians have also used ozone therapy as an adjuvant treatment to the main clinical or pharmacological approaches [36,37,38,39,40].

We have found two previous different revisions on treatment of NG. The first was published in 2016 [27] and proposed the use of an adapted version of the protocol used for adult patients to treat young ones. The second one was published in 2018 [25] and aimed to highlight the medical rationale behind the prescription of antibiotic drugs to manage orofacial infection (including NG) in paediatric outpatients.

This scoping review aims to detail the extent and types of symptoms related to the treatment of necrotizing gingivitis in paediatric patients. Furthermore, a decision tree protocol was developed for the management of NG in paediatric patients with or without lymphadenopathy, fever, or other systemic clinical manifestations commonly associated with NG. This approach was taken in order to underscore the necessity of adopting a personalized treatment strategy.

## 2. Materials and Methods

### 2.1. Type of Study

The proposed scoping literature review follows the stages indicated by the Preferred Reporting Items for Systematic Reviews and Meta-Analysis (PRISMA). The protocol was registered in the Open Science Framework on March 2024 (DOI:10.17605/OSF.IO/FGHJD).

This phase of the study was conducted in February 2024 using different electronic databases, utilising the following mesh terms: “Gingivitis, Necrotizing Ulcerative/drug therapy”, “Gingivitis, Necrotizing Ulcerative/therapy”, “Adolescents”, and “Children”, or the age filter “Child: birth—18 years”.

### 2.2. Review Question

The following PCC framework was used to develop the research question: (1) Population: paediatric patients with a necrotizing gingivitis diagnosis; (2) Concept: treatment of NG in a paediatric population; (3) Context: not applicable (N/A). Thus, the review question was as follows: “What is the protocol of treatment of necrotizing gingivitis in paediatric patients”?

### 2.3. Inclusion and Exclusion Criteria

This review includes any type of studies, including case–control studies, clinical studies, cross-sectional studies, prospective studies, randomized controlled trials, and reviews about the treatment of paediatric patients with a necrotizing gingivitis diagnosis.

The following reports were excluded: studies that did not propose a treatment protocol for NG, studies that included adult patients older than 18 years, and studies with unavailability of full text versions. No publication date restrictions were applied.

### 2.4. Search Strategy and Study Selection

Studies were searched on the electronic databases Lilacs, Cochrane, JBI Evidence Synthesis, PubMed, Scopus, and Web of Science. A combination of controlled descriptors or mesh terms guided the search strategy, without the application of any restrictions of language or date of publication.

Reading titles and abstracts allowed the early selection of the studies based on the eligibility criteria. Two reviewers, in an independent and blinded manner, performed the entire review from the early stages to the full text reading of the selected studies.

### 2.5. Data Collection

The useful data extracted from the selected studies were collected in an Excel table (Table 1), reporting the following: (1) authors and year of publication; (2) study design; (3) patient gender; (4) smoking habits; (5) systemic diseases; and (6) age of patients, as well as the type of treatment, such as (7) mechanical therapy for the debridement of soft and hard deposits; (8) local antiseptics; (9) antibiotic therapy; and (10) follow-up.

### 2.6. Risk of Bias

Following the manual published by the Joanna Briggs Institute, this scoping review cannot be interpreted as risking any bias or quality assessment, since our aim was to identify knowledge gaps in the topic of our interest [41].

## 3. Literature Review Results

**Figure 1:**
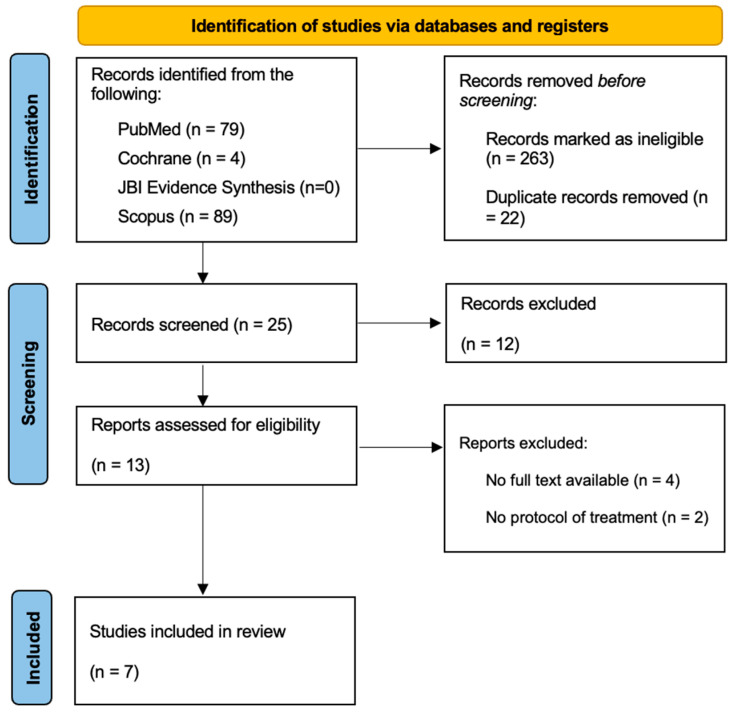
PRISMA flowchart on the selection and evaluation of scientific articles.

A total of 310 articles were initially selected; of these, 25 were included for abstract reading after the removal of duplicates. Seven studies (two case reports, three guidelines, and two reviews) were finally considered, because they fulfilled all the inclusion criteria: (1) articles about NG treatment; (2) studies involving paediatric patients; (3) articles with full text available. The findings were presented in detail and listed in the flow diagram established by the Preferred Reporting Items for Systematic Reviews and Meta-Analysis (PRISMA) method (Figure 1).

The results are detailed in Table 1. In total, 86% of the studies agree that the initial treatment for NG is the removal of irritants, specifically the bacterial plaque and calculus deposits [9,14,23,24,25,26]. The studies recommend that debridement should begin supragingivally, and progress subgingivally [9,14] with a gentle approach [9,25] due to the painful symptoms typically experienced by the patients at this stage. All the selected studies, except for one [24], recommend the use of local antiseptics such as chlorhexidine [9,14,26] or hydrogen peroxide [25], or a combination of both [23,27]. Regarding antibiotic therapy, metronidazole is the preferred choice in 71% of the studies [9,23,24,26,27]. Only one study recommends the use of amoxicillin [25], while another study does not include any antibiotic treatment protocol [14]. In total, 71% of the papers suggest that antibiotic therapy should be prescribed when systemic signs and symptoms are present [9,23,24,25,27]. A straight follow-up is recommended in 71% of the articles [9,14,23,24,27].

## 4. Clinical Cases

### 4.1. Case 1

A 15-year-old female subject presented in the Department of Odontostomatology of the “Bambino Gesù” Children’s Hospital (Rome) with oral pain, inappetence, spontaneous gingival bleeding, swollen lips, and fever.

**Figure 2:**
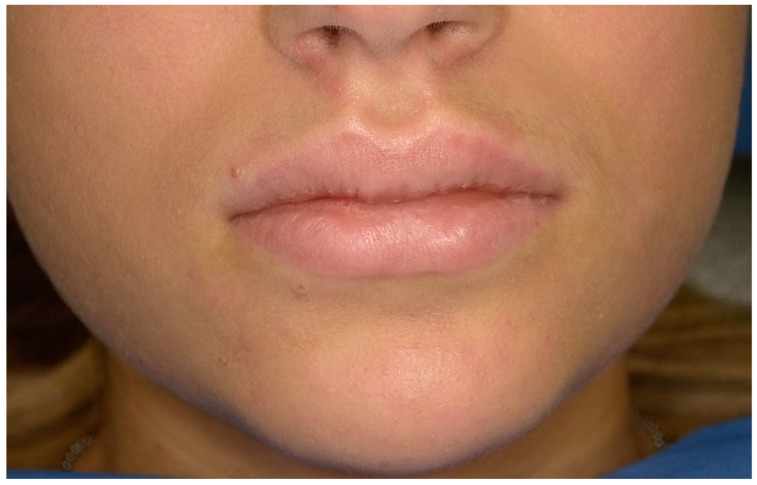
Case 1. An extraoral photograph revealing the presence of swelling in the lips and the perioral area.

**Figure 3:**
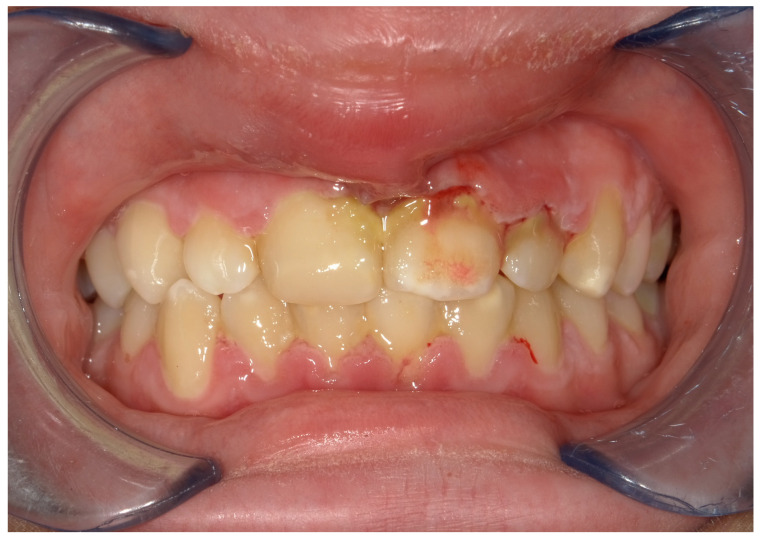
An intraoral photograph demonstrating the presence of purulent exudate in the areas between teeth #11 and #23, and #42 and #32; spontaneous bleeding; and gum hypertrophy.

From an extraoral examination, the patient presented lymphadenopathy and swelling of the lips and the area around the mouth (Figure 2), making intraoral photography difficult (Figure 3). From an intraoral examination, the girl presented purulent exudate in the areas between teeth #11 and #23, and #42 and #32 JGJ 84-2008 [42], as well as spontaneous bleeding and gum hypertrophy (Figure 3). Foetor ex ore was also found.

**Figure 4:**
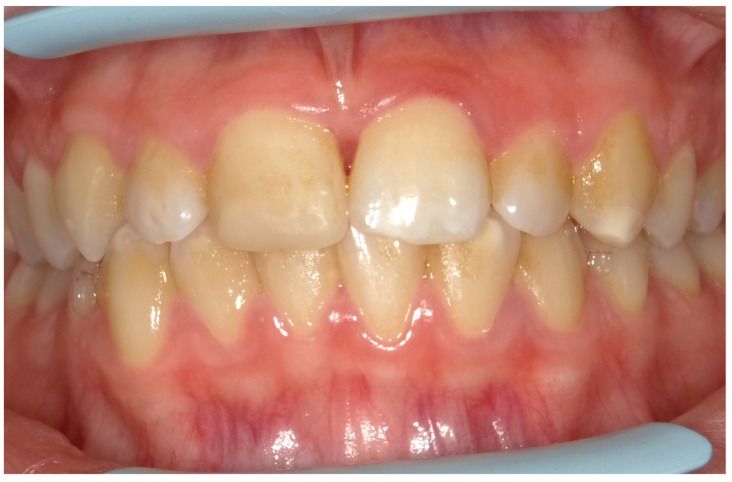
An intraoral photograph taken five days post-initiation of antibiotic therapy. The soft tissue is exhibiting signs of healing.

**Figure 5:**
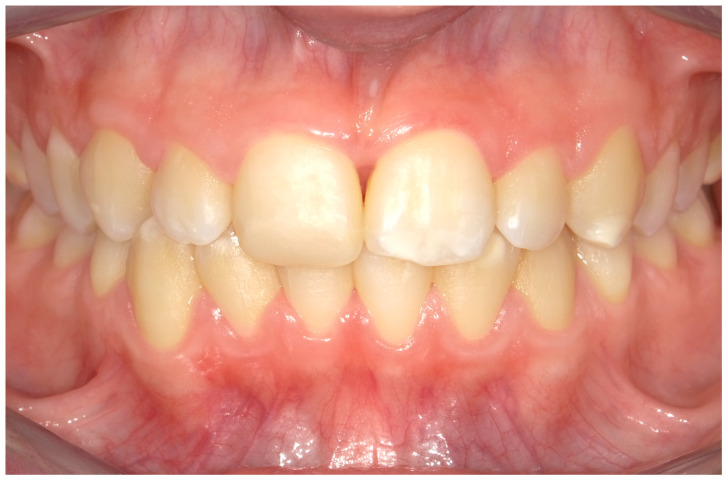
An intraoral photograph taken after one week. The soft tissue appears to be healthy.

**Figure 6:**
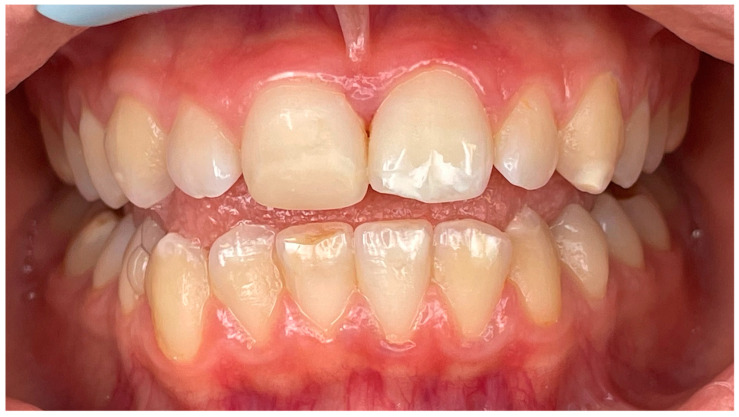
Intraoral photo obtained at one-year follow-up appointment.

Given the pain and the presence of systemic involvement of the patient, the removal of soft and hard deposits was postponed until after five days of antibiotic therapy with 250 mg of metronidazole, to be taken orally every 8 h for five days. Then, the patient was re-evaluated five days later (Figure 4), and professional oral hygiene was performed in addition to decontamination of the oral mucosa by irrigation with hydrogen peroxide. The patient was taught proper oral hygiene at home, using a soft-bristled electric brush and a hydrogen peroxide-soaked brush to clean and decontaminate interdental spaces. In addition, the girl was prescribed 0.2% chlorhexidine mouthwash for one week (two rinses a day). Thus, the patient was re-evaluated after one week (Figure 5) and ozone therapy was performed on the soft tissue, following the instructions of the ozone generator producer (Ozone DTA—Sweden & Martina). Ozone therapy was repeated a total of three times every two days. At home, after the first week of chlorhexidine mouthwash, the patient switched to an ozonated oil-based mouthwash. The other cleaning instruments remained unchanged. To avoid recurrence, the patient was re-evaluated once a month for the first six months, and after one year, the soft tissue’s condition appeared healthy (Figure 6).

### 4.2. Case 2

**Figure 7:**
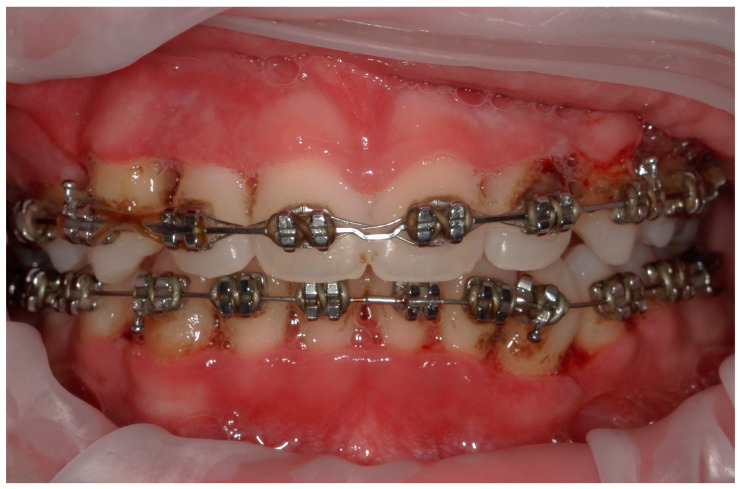
Case 2. An intraoral photograph demonstrating the presence of spontaneous bleeding, gum hypertrophy, and the appearance of “punched-out” interdental papillae.

**Figure 8:**
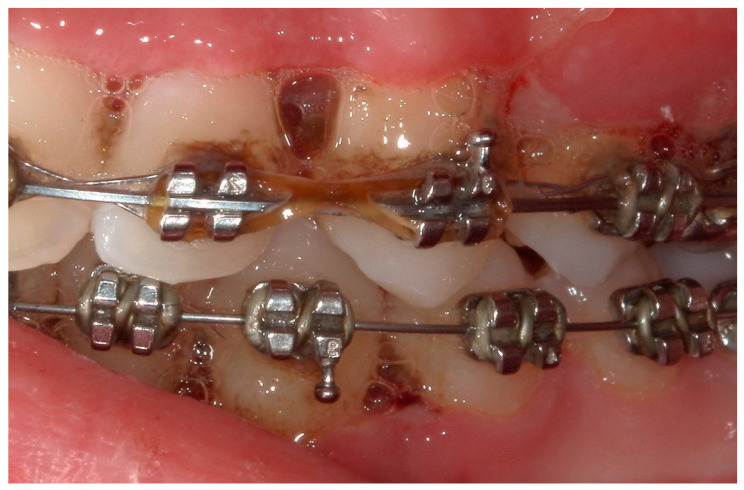
Detail of the “punched-out” interdental papillae.

**Figure 9:**
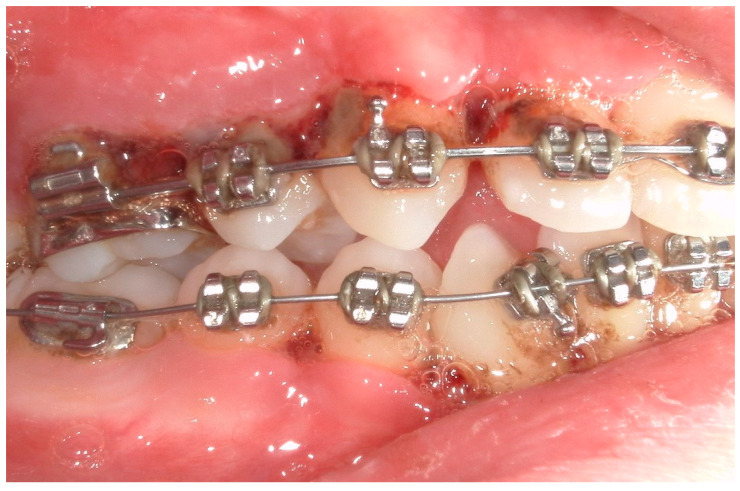
A lateral photograph of the oral cavity with visible purulent exudate, bleeding, and gum hypertrophy.

**Figure 10:**
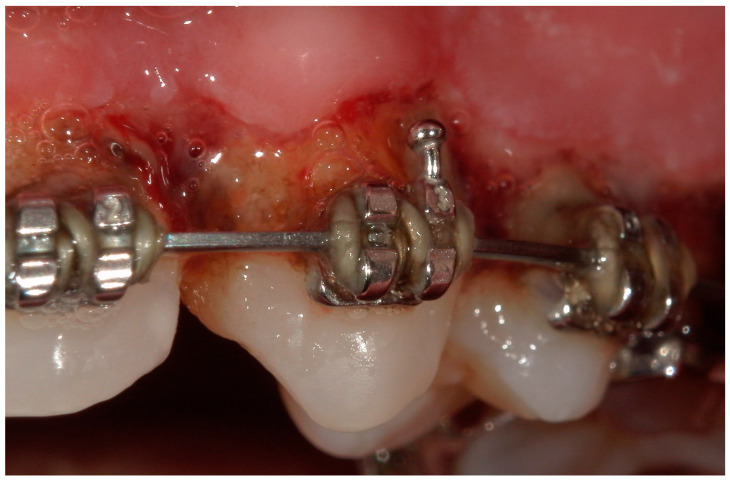
A lateral intraoral photograph taken after one week. The soft tissue appears to be in good condition.

**Figure 11:**
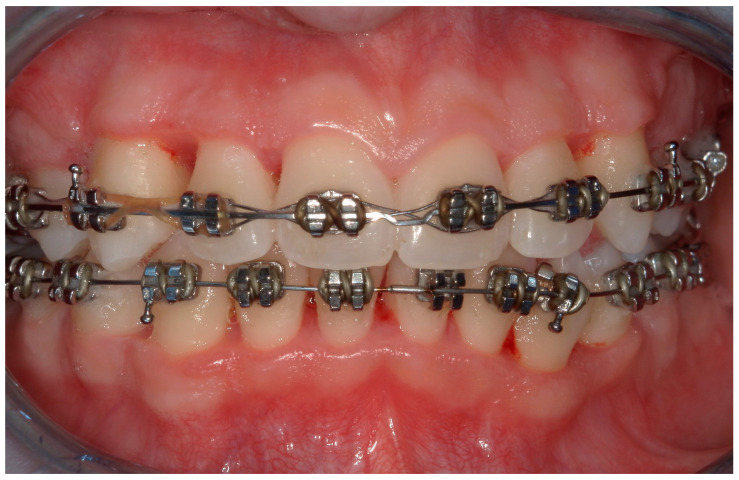
An intraoral photograph taken after one week.

**Figure 12:**
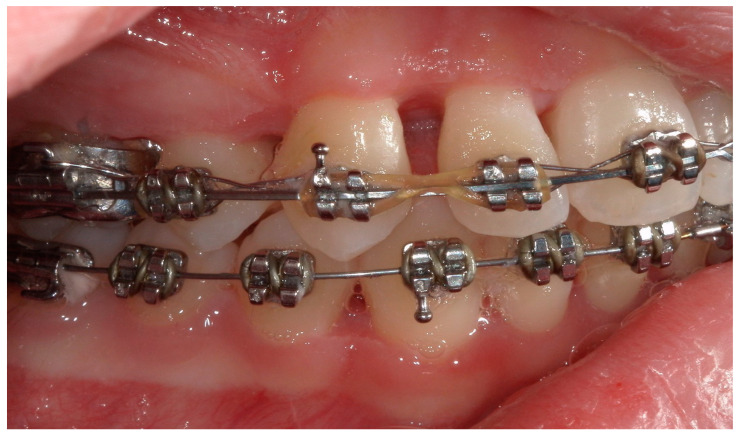
A lateral intraoral photograph taken after one week. The soft tissue appears to be in good condition.

**Figure 13:**
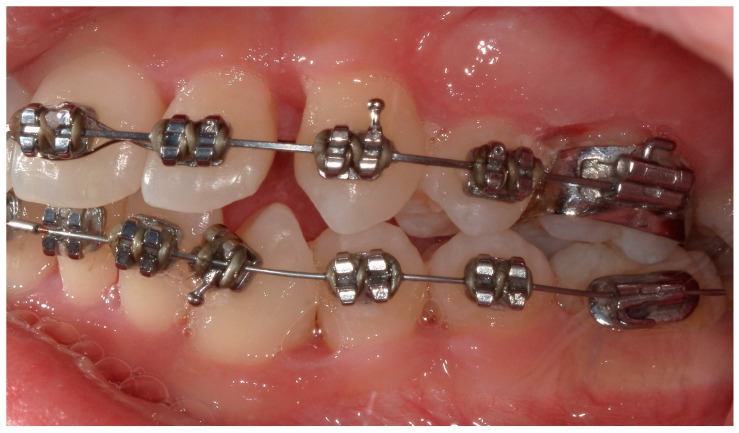
A lateral intraoral photograph taken after one week. The soft tissue continues to look healthy.

**Figure 14:**
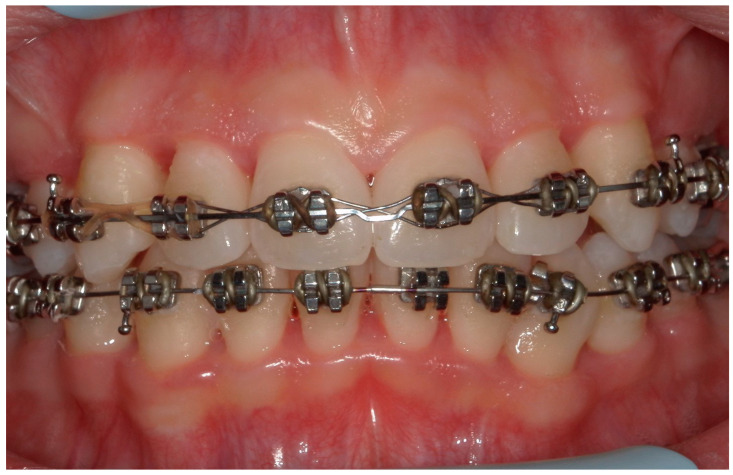
An intraoral photograph of the mouth taken after six months. The soft tissue is visibly healthy.

**Figure 15:**
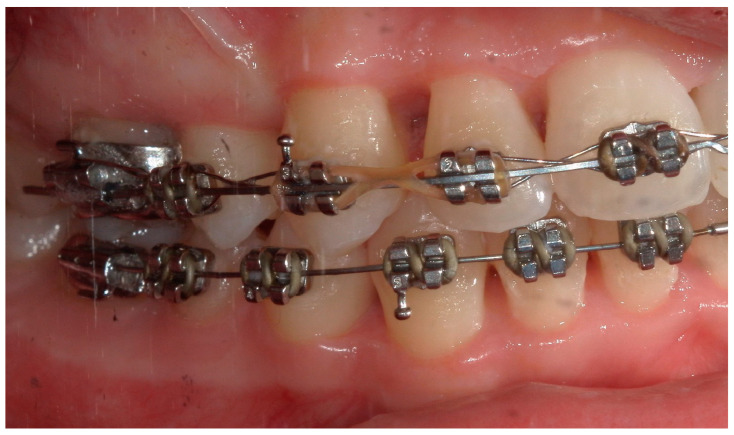
A lateral intraoral photograph taken after six months. The soft tissue appears to be in good condition.

**Figure 16:**
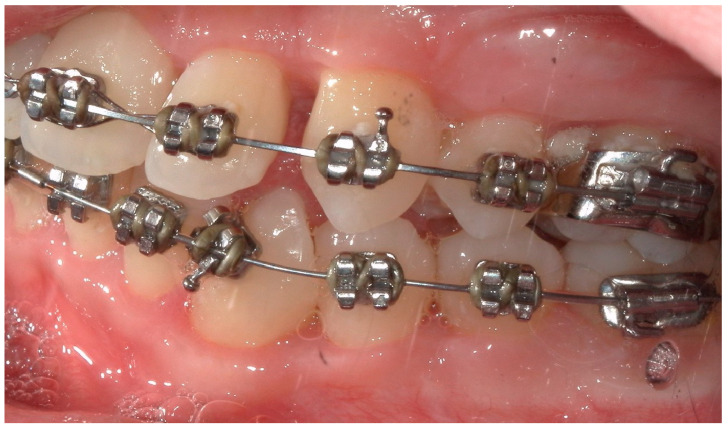
A lateral intraoral photograph of the mouth taken after six months. The appearance of the soft tissue is healthy.

A 15-year-old female subject, undergoing orthodontic multibrackets treatment, presented in the Department of Odontostomatology of the “Bambino Gesù” Children’s Hospital (Rome) with oral pain and nightly spontaneous gingival bleeding. From an extraoral examination, the patient had no lymphadenopathy or other systemic clinical signs. From an intraoral examination, the girl presented mild purulent exudate, spontaneous bleeding, gum hypertrophy, and the appearance of “punched-out” interdental papillae (Figure 7, Figure 8, Figure 9 and Figure 10). Foetor ex ore was also found. Given the absence of systemic involvement of the patient, professional oral hygiene was performed without antibiotic therapy in addition to decontamination of the oral mucosa by irrigation with hydrogen peroxide. The patient was taught proper oral hygiene at home, using a soft-bristled electric brush and a hydrogen peroxide-soaked brush to clean and decontaminate interdental spaces. In addition, the girl was prescribed 0.2% chlorhexidine mouthwash for one week (two rinses a day). Thus, the patient was re-evaluated after one week (Figure 11, Figure 12 and Figure 13) and ozone therapy was performed on the soft tissue, following the instructions of the ozone generator producer (Ozone DTA—Sweden & Martina). Ozone therapy was repeated a total of three times every two days. At home, after the first week of using chlorhexidine mouthwash, the patient switched to an ozonated oil-based mouthwash. The other cleaning instruments remained unchanged. To avoid recurrence, the patient was re-evaluated once a month for the first six months, and after one year, the soft tissue’s condition appeared healthy (Figure 14, Figure 15 and Figure 16).

## 5. Discussion

The acute inflammatory process affecting the gingival tissues that characterizes NG is sometimes associated with chronic and severely compromising conditions weakening the host’s immune system, such as severe malnourishment in children, extremely poor living conditions, and more than moderate infections, especially in temporarily and/or moderately compromised patients, including tabagists, adults under psycho-social stress, and HIV patients [1].

In the literature, the research available on this topic is low in high-quality data [5], especially for paediatric patients, for whom the literature typically adapts adult treatment protocols [27]. As professionals aiming to manage oral health in paediatric patients, dentists, as well as other professionals like dental hygienists, may frequently encounter conditions that require a more personalized and specialized approach [43,44]. As observed by different authors, when a dentist or a dental hygienist is faced with a rare condition, the availability of official and unequivocal treatment paths represents a necessity and a mandatory reference that must be known and applied clinically for both ethical and legal reasons [44,45].

In this scoping review, we aim to detail a decision tree protocol for the management of NG in paediatric patients, focusing on differences in the choice of antibiotic therapy; we also propose treatment protocols that are specific to the presence or absence of systemic manifestations and suggest ozone therapy as an adjunctive treatment.

From the articles reviewed, we can affirm that first we should divide NG patients into two categories: patients with lymphadenopathy, pain, malaise, high levels of purulent exudate, or other systemic signs and symptoms, and patients without systemic involvement. In the treatment of patients with systemic signs and symptoms, it is strongly recommended to use systemic antibiotic therapy [9,23,24,25,27] to avoid bacteraemia, which could be caused by the high presence of bacteria in the mouth and by necrotizing gingival tissues [27,46]. Patients without systemic involvement may proceed directly to the second phase of treatment: periodontal therapy, including the removal of bacterial plaque and tartar to eliminate irritants and prevent disease progression. Debridement can be also performed under pain control induced by anaesthesia (from local to general anaesthesia including conscious sedation) [27] if necessary. Subgingival gentle rinsing with hydrogen peroxide could be also used. Oxygen-releasing agents, in fact, contribute to mechanical cleaning and provide an antibacterial effect against anaerobes induced by the oxygen [10,25]. In the literature, experiences of local oxygen therapy have been documented. In 2006, Gaggl and coworkers showed that treatment of acute necrotizing periodontal disease with the aid of adjunctive oxygen therapy could result in the reduction of periodontal tissue damage, and this corresponds to the early eradication of pathogenic anaerobes [47].

The third phase of treatment is at-home therapy, with local antiseptic mouthwashes such as chlorhexidine gluconate [9,14,23,26], or local application of chlorhexidine-based gel [27]. It is also possible to implement the use of oxygen-releasing agents, such as hydrogen peroxide, in the form of rinsing [23,25] or gel application [27], or local decontamination of the oral cavity with gauze soaked in H_2_O_2_ [27]. Meticulous brushing with a soft bristle toothbrush [23] and the use of dental floss [26] are essential. Most studies have also recommended careful follow-ups [9,14,23,26,27] for at least 6 months, in order to prevent recurrences.

The results of this review have led to the conceptualization of a decision tree protocol for the treatment of necrotizing gingivitis, which is presented below. Initially, a clinical examination is conducted to determine whether the patient is exhibiting any systemic involvement. In cases of systemic involvement, the administration of antibiotic therapy is indicated with 250 mg of metronidazole every 8 h (5 days), and the periodontal therapy will be postponed. The periodontal therapy includes gentle mechanical removal of hard and soft deposits and decontamination with hydrogen peroxide. This phase is associated with home treatment. For the first two weeks, it is recommended that the patient rinses with a chlorhexidine-based mouthwash and uses a manual or electric (depending on pain) soft-bristled toothbrush. It may also be helpful to decontaminate the interproximal spaces that may have formed because of gingival resorption with a pipe cleaner soaked in hydrogen peroxide. It is recommended that a re-evaluation of the patient be conducted one week after the initial assessment. If necessary, periodontal therapy may be repeated at this stage and ozone therapy of the soft tissues may also be administered. After 1–3 days, ozone therapy should be repeated a total of three times.

Ozone therapy is also effective as an adjunctive treatment of periodontal diseases [48], although the literature regarding this support is not consistent. However, previous cases have demonstrated the successful and safe use of ozone therapy [36,37,38,39]. In the periodontal therapy phase, it is possible to add the topical treatment of ozone therapy. Its use is justified because it has bactericidal properties and stimulates blood circulation with an increased glycolysis process rate in the red blood cells. Consequently, the amount of oxygen released to the tissues tends to significantly increase. Moreover, ozone stimulates the activation of the immune response [28,29,30,31,32,33].

At home, after two weeks of chlorhexidine mouthwash, it is recommended to use an ozonated oil-based daily mouthwash to support professional ozone therapy. It is also recommended to re-evaluate the patient at close intervals based on the severity of the disease.

In summary, the protocol should consist of the following phases: (1) evaluation of systemic signs and symptoms related to the disease; (2) in case of systemic involvement, prescription of antibiotic therapy, with 250 mg of metronidazole every 8 h (5 days); (3) gentle mechanical removal of hard and soft deposits using ultrasonic and/or manual devices; (4) decontamination of the oral mucosa by irrigation with hydrogen peroxide; (5) ozone therapy of soft tissue.

**Figure 17:**
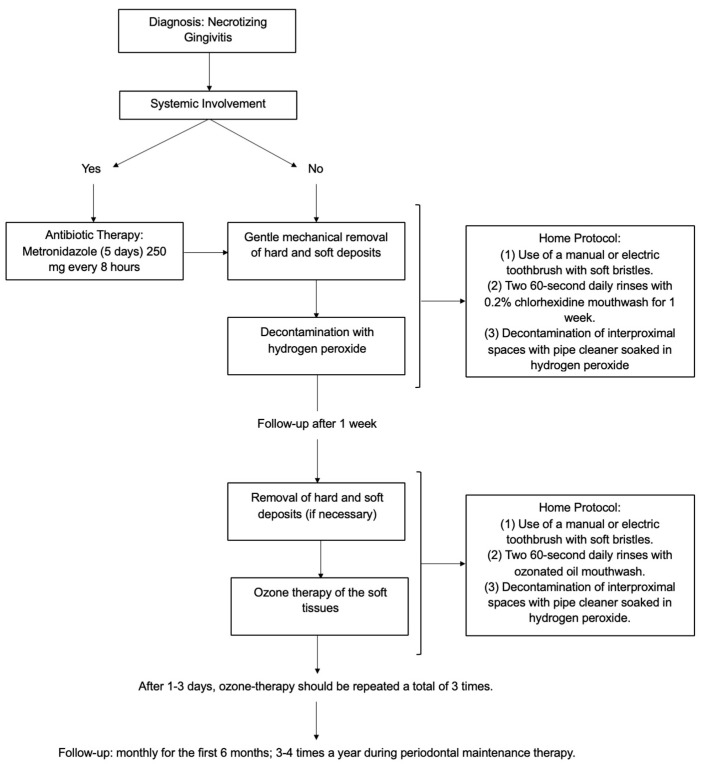
Flowchart of the proposed treatment protocol.

This professional treatment should be supported by home therapy, which includes (1) the use of a manual or electric toothbrush with soft bristles; (2) two 60 s daily rinses with 0.2% chlorhexidine mouthwash for two weeks; (3) decontamination of interproximal spaces with a pipe cleaner soaked in hydrogen peroxide. The patients should be re-evaluated after 1 week. At this appointment, the following steps are carried out: (1) removal of hard and soft deposits (if necessary); (2) ozone therapy of the soft tissue. Home protocol: (1) the use of a manual or electric toothbrush with soft bristles; (2) two 60 s daily rinses with ozonated oil mouthwash; (3) decontamination of interproximal spaces with a pipe cleaner soaked in hydrogen peroxide. After 1–3 days, ozone therapy should be repeated a total of three times. The patients in the cases we studied went to a monthly follow-up appointment during the first 6 months; the periodontal maintenance therapy then allowed the extension of the follow-up to 3–4 times a year (Figure 17).

The presented protocol comprises distinct treatment phases, with the objective of enabling the protocol to be tailored to the specific characteristics of each patient. Specifically, the treatments are based on the patient’s response over time, initially focusing on the presence or absence of systemic involvement and/or odontalgia, and then monitoring the healing response over time.

The current review presents two limitations. First, the number of studies included in the review is limited, resulting in a narrow scope of analysis. Second, the studies yield inconsistent results. A larger number of patients should be included in future studies to enhance the precision of the findings.

## 6. Conclusions

This review outlines the lack of research on the treatment of NG in paediatric patients. The treatment protocol proposed in this paper was effective in treating NG, both in combination with and without the use of antibiotic therapy. In fact, the latter’s use in dentistry can be reduced by assessing the clinical condition of each patient and the type of treatment that needs to be performed. This approach contributes to the reduction of the risk of antibiotic resistance without compromising clinical success, and it aligns with the modern concept of personalized medicine. Moreover, the use of ozone therapy as a supportive therapy in the treatment of NG can be a valuable tool in the healing of oral tissues. Of course, further properly defined randomized trials with different therapeutic approaches and larger sample sizes are needed.

## Figures and Tables

**Table 1:** Data collection for the selected study.

Author, Year	Study Design	Gender	Smoke (Yes/No)	Systemic Diseases	Age	Mechanical Therapy	Local Antiseptics	Antibiotics	Follow-Up
Sangani, 2013 [9]	Case Report	3 female, 1 male	2 no, 2 yes	No	15/18 yo	Classic signs: local debridement Mild signs: local debridement	Classic signs: chlorhexidine mouthwash Mild signs: chlorhexidine mouthwash	Classic signs: metronidazole tds 200 mg 3 days Mild signs: /	Classic signs: review 1 week Mild signs: review 1–2 week
Martos, 2018 [14]	Case Report	Male	No	No	18 yo	Initial phase of supragingival hard and soft deposits removal and successive subgingival scaling.	Chlorhexidine gluconate 0.12%: mouth rinse twice a day for 30 days.	N/A	Weekly throughout treatment, monthly for the first 6 months post-treatment, and 2–3 times a year during the periodontal maintenance therapy.
Studen-Pavlovich, 2006 [23]	Guideline	N/A	N/A	N/A	Adolescents	Deplaquing and debridement with ultrasonic scaler with water spray to minimize discomfort to the patient.	Brushing with a soft bristle toothbrush. Rinse with a 1.5% hydrogen peroxide or a 0.2% chlorhexidine gluconate solution.	Fever and systemic symptoms: penicillin or metronidazole.	Advised
Califano, 2003 [24]	Guideline	N/A	N/A	N/A	Children and Adolescents	Mechanical debridement with ultrasonics.	N/A	If the patient is febrile: metronidazole and penicillin.	Advised
Dar-Odeh, 2018 [25]	Review	N/A	N/A	N/A	Children and Adolescents	Gentle deplaquing and debridement	Antiseptic mouth washes and oxygen–releasing agents.	Systemic signs and symptoms: Amoxicillin (3 days): Children > 3 months and <40 kg: 20–40 mg/kg/day in divided 8 hourly doses OR 25–45 mg/kg/day in divided 12 hourly doses. Children > 40 kg: 250–500 mg 8 hourly OR 500–875 mg 12 hourly.	N/A
Bermejo-Fenoll, 2004 [26]	Guideline	N/A	N/A	N/A	Children and Adolescents	Surgical and mechanical debridement of the gingival lesions.	Mouth rinses with 0.12% chlorhexidine. Brushing and dental floss.	Intense pain: minor analgesics. Metronidazole: 250 mg every 8 h for 10 days. Other antibiotics: penicillin and clindamycin and amoxicillin/ clavulanic acid.	N/A
Marty, 2016 [27]	Review	N/A	No	N/A	Children	Postponed mechanical therapies to avoid bacteremia. Debridement under local anesthesia and conscious sedation or general anesthesia.	Gel with chlorhexidine and 3% H_2_O_2_ or topic use of 3% H_2_O_2_ diluted by half with water. Application of sterile compresses dipped in hydrogen peroxide.	General involvement: three divided doses of metronidazole from 30 to 50 mg/kg/day for 7–10 days.	Regularly, for at least 1 year.

## Data Availability

The protocol was registered in the Open Science Framework on 26 March 2024 (DOI:10.17605/OSF.IO/FGHJD).
